# Criminal Responsibility in Individuals with Gambling Disorder in Brazil

**DOI:** 10.1007/s10899-026-10474-7

**Published:** 2026-02-18

**Authors:** Thiago Henrique Roza, Daniel Tornaim Spritzer, Clarice Sandi Madruga, Felix Henrique Paim Kessler, Lisieux Elaine de Borba Telles

**Affiliations:** 1 Department of Forensic Medicine and Psychiatry, Universidade Federal do Paraná, Rua Padre Camargo, 280, Curitiba, 80060-240 PR Brazil; 2 Graduate Program in Psychiatry and Behavioral Sciences, Federal University of Rio Grande do Sul (UFRGS), Porto Alegre, RS Brazil; 3 Laboratory of Digital Psychiatry, Federal University of Paraná (UFPR), Curitiba, PR Brazil; 4 National Institute of Science and Technology in Digital Mental Health (INCT–SMD), Porto Alegre, RS Brazil; 5 Department of Psychiatry, Federal University of São Paulo (UNIFESP), São Paulo, SP Brazil; 6 Associação Paulista para o Desenvolvimento da Medicina (SPDM), São Paulo, SP Brazil; 7 Department of Psychiatry and Legal Medicine, Federal University of Rio Grande do Sul (UFRGS), Porto Alegre, RS Brazil

Gambling disorder is an addictive psychiatric disorder characterized by problematic, recurrent, and persistent patterns of gambling, which consists of playing with something at risk, in the hopes of receiving more value in return. Gambling behavior can take several formats and expressions, including casino involvement, lotteries, poker games, sports betting, and other forms of online gambling (American Psychiatric Association, 2022; Potenza et al., 2019). Gambling disorder is associated with significant distress, poor quality of life, and impairment in several domains. Furthermore, gambling disorder is often linked with several negative outcomes, such as marital and relationship problems, depression, suicidality, sleep difficulties, medical symptoms and conditions, as well as social and financial problems (Potenza et al., 2019).

A recent systematic review and meta-analysis reported that, globally, 46.2% (95% CI: 41.7–50.8) of adults had engaged in some form of gambling in the previous year, corresponding to approximately 2.3 billion individuals (Tran et al., 2024). Among adults, 8.7% (95% CI: 6.6–11.3) engaged in risky gambling behavior, and 1.41% (95% CI: 1.06–1.84) met criteria for problematic gambling. Higher proportions were observed among men, both in gambling participation (49.1% vs. 37.4% for women) and problem gambling (2.2% vs. 1.0% for women). Online casinos and slots gambling were associated with the highest proportion of problematic use 15.8% (95% CI: 10.7–21.6) (Tran et al., 2024). A rise in online gambling was also documented, with adult participation increasing from 5.5% before 2016 to 10.0% after 2020 (Tran et al., 2024). These findings reinforce concerns about the growing burden of gambling-related harm and highlight the need for public health responses tailored to high-risk gambling types and vulnerable groups (Calado & Griffiths, 2016; Reith et al., 2019).

Gambling regulations in Brazil have undergone substantial changes in recent years. In 2018, the approval of Law No. 13,756/2018 provided preliminary authorization for sports betting (LEI N^o^ 13.756/2018, (LEI 2018); Secretaria de Comunicação Social, 2024). Even though Brazil has experienced periods of varying degrees of approval and prohibition of gambling throughout its history, prior to the enactment of the 2018 law most forms of gambling were illegal in the country. At that time, only state lotteries, horse race betting, and certain forms of poker were legally permitted. Nevertheless, despite these prohibitions, gambling remained highly prevalent, with numerous gambling venues operating illegally across the country (Tavares, 2014). In 2023, the Brazilian government enacted Law No. 14,790/2023, which further regulated provisions introduced by the 2018 law, including the regulation of online gambling (Bairros, 2025, LEI N^o^ 14.790/2023, 2023; Secretaria de Comunicação Social, 2024). Following the enactment of the 2023 law, the Brazilian government, through the Secretariat of Prizes and Betting of the Ministry of Finance, established several mechanisms to regulate gambling operations, advertising, and gambling participation in the country. These measures included policies aimed at preventing money laundering and fraud, as well as the prohibition of multiple gambling companies from operating in Brazil. Importantly, children and adolescents (individuals under 18 years of age) remain prohibited from engaging in gambling activities in Brazil (Bairros, 2025, LEI N^o^ 14.790/2023, 2023; Secretaria de Comunicação Social, 2024).

In Brazil, recent findings from the third edition of the National Survey on Alcohol and Drugs (LENAD III) show that 25.9% (95% CI: 23.8–28.1) of individuals aged 14 and older had gambled at least once in their lifetime, and 17.6% (95% CI: 16.0–19.4) had done so in the previous year, corresponding to approximately 28 million Brazilians (UNIFESP, 2025). Based on the Problem Gambling Severity Index (PGSI), 6.5% of the population were classified as low- or moderate-risk gamblers (PGSI 1–7), while 0.8% (95% CI: 0.5–1.3) met criteria for problem gambling (PGSI ≥8). Although men showed higher overall gambling participation rates, adolescents emerged as a particularly vulnerable group: 55.2% of adolescents who gambled in the previous year exhibited either at-risk or problem gambling patterns. Engagement with digital sports betting platforms was associated with substantially higher levels of harm, with 66.8% (95% CI: 59.0–73.9) of users classified as at-risk or problem gamblers, compared to 26.8% among users of other gambling modalities (UNIFESP, 2025).

According to the Article 26 of the Brazilian Penal Code, individuals who commit criminal offenses under the context of psychiatric symptoms may be subjected to a forensic psychiatric evaluation to retrospectively assess their capacity for understanding and self-determination in light of the psychiatric symptomatology present at the time of the offense. Individuals whose capacity to understand the unlawful nature of the act and/or to act in accordance with such understanding is completely impaired due to psychiatric symptoms are considered “legally insane” and criminally non-imputable. An example of criminal non-imputability is an individual with schizophrenia charged with homicide committed in the context of active psychotic symptoms (e.g., paranoid delusions and auditory hallucinations), in which the victim is directly related to the content of the psychotic experiences. In this context, the individual would lack the capacity for understanding and self-determination at the time of the offense (Abdala-Filho et al., 2016, Código Penal Brasileiro, 2023).

In cases in which individuals exhibit partial impairment of their capacity for understanding and/or self-determination due to psychiatric symptoms, they are considered to have diminished criminal responsibility (i.e., to be semi-imputable) at the time of the offense. This may result in a reduction of the sentence by one to two-thirds or in the substitution of the sentence with a security measure. In cases involving serious crimes, the application of a security measure may involve compulsory psychiatric treatment under involuntary inpatient confinement (Abdala-Filho et al., 2016, Código Penal Brasileiro, 2023).

Globally, there is ongoing debate regarding the extent to which gambling disorder may impair an individual’s capacity for self-determination at the time of a criminal offense, a discussion that also extends to the Brazilian legal context. For example, the text-revised fourth edition of the Diagnostic and Statistical Manual of Mental Disorders (DSM-IV-TR) included engagement in illegal activities to finance gambling as a distinct diagnostic criterion for pathological gambling (American Psychiatric Association, 2000; Grant & Potenza, 2007; Mestre-Bach et al., 2018). In the DSM-5, this criterion was removed following substantial criticism, including the argument that criminal offenses committed to finance gambling should be regarded as indicators of disorder severity rather than as a distinct and independent diagnostic criterion for gambling disorder (American Psychiatric Association, 2013; Mestre-Bach et al., 2018).

Gambling disorder, as a behavioral addiction, is associated with impaired self-control, heightened impulsivity, risk-taking behaviors, and a tendency to seek immediate reward or relief with limited consideration of longer-term consequences (Mestre-Bach et al., 2018). In some cases, individuals with gambling disorder may engage in criminal behaviors directly associated with gambling involvement, including fraud, theft, forgery, or robbery (Blum & Grant, 2017; Grant & Chamberlain, 2023; Mestre-Bach et al., 2021). For instance, among treatment-seeking young adults (*n* = 808) with gambling disorder, 36% had a history of gambling disorder-related illegal offenses (Mestre-Bach et al., 2021). Criminal behavior among individuals with gambling disorder is frequently linked to financial motives, particularly those related to sustaining gambling activities or attempting to recover monetary losses resulting from gambling involvement (Grant & Chamberlain, 2023; Mestre-Bach et al., 2021).

On the one hand, the association between gambling disorder and criminal behavior is complex and likely influenced by multiple additional factors, including sociodemographic and socioeconomic characteristics, psychiatric comorbidities, personality traits, and gambling-related life circumstances (such as the presence and magnitude of financial debt), among others (Mestre-Bach et al., 2018). In this context, considerable proportions of people with gambling disorder present conduct problems and a history of criminal behaviors that are more closely associated with comorbid psychiatric conditions, such as antisocial personality disorder, or that are not directly associated with gambling behaviors, including violent and drug-related offenses (Blum & Grant, 2017; Grant & Chamberlain, 2023). In such cases, the individual should not be considered fully or even partially criminally non-imputable, and the diagnosis of gambling disorder per se cannot be regarded as a criterion for mitigating criminal responsibility (Blum & Grant, 2017).

**Fig. 1:**
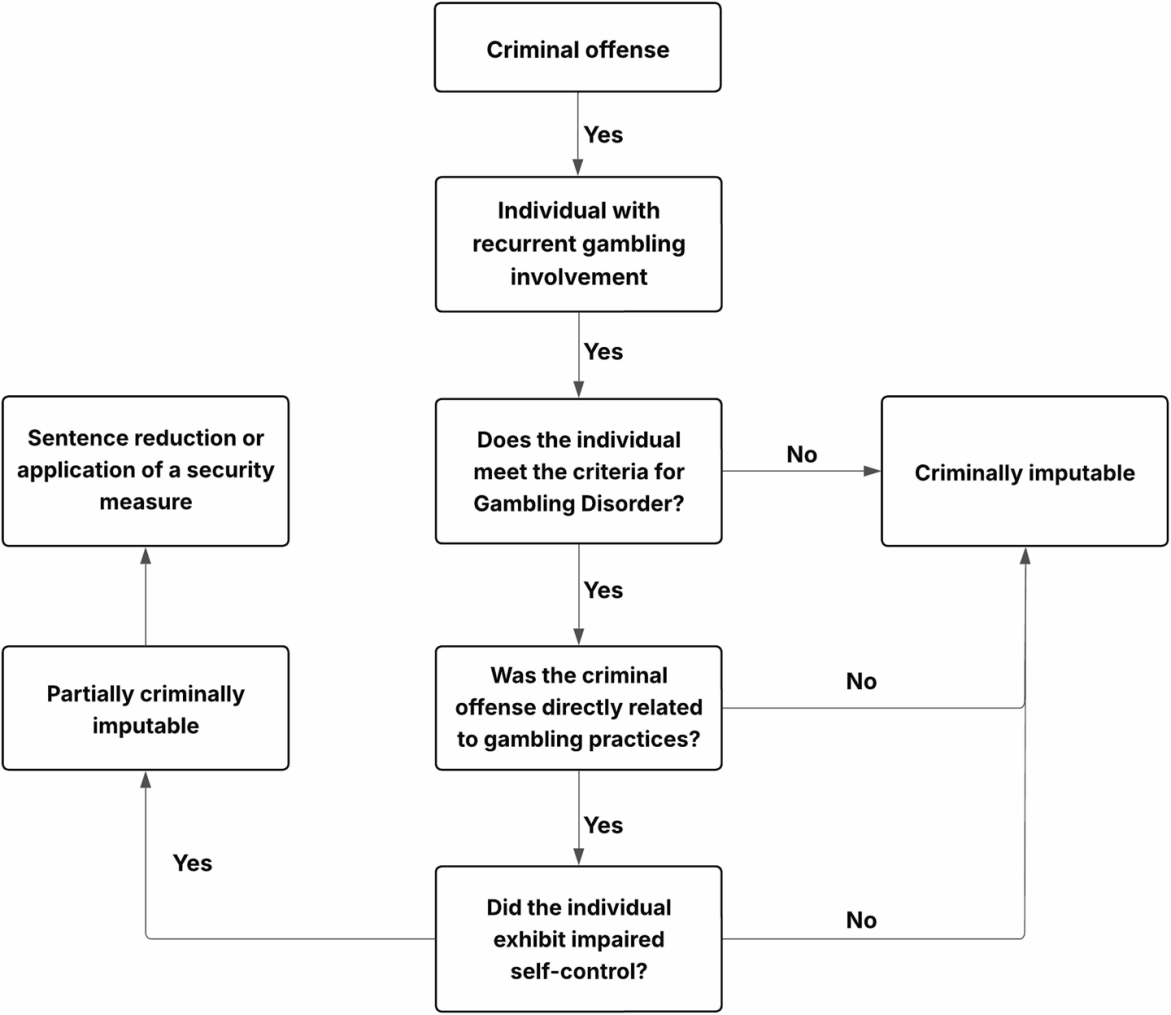
Flowchart for the interpretation of criminal responsibility in individuals with gambling disorder in Brazil. It is important to note that, although gambling disorder is associated with cognitive distortions, such as the illusion of control, there is no evidence to suggest that gambling disorder per se is associated with an impaired capacity to understand the consequences or the unlawful nature of criminal behavior. This capacity is a distinct criterion for diminished criminal responsibility under Brazilian law and is typically compromised in conditions such as psychotic or major neurocognitive disorders (Abdala-Filho et al., 2016; Mestre-Bach et al., 2018). Therefore, in the assessment of criminal responsibility in these individuals, reduced self-control is more likely to be observed specifically in relation to gambling-associated illegal acts. The presence of a prior criminal history (preceding the onset of gambling disorder), criminal behaviors unrelated to gambling practices, and comorbid psychiatric conditions (such as antisocial personality disorder) should be carefully evaluated whenever possible, as these factors argue against a finding of semi-imputability. Another important factor to consider is the occurrence of criminal behavior in contexts where financial resources are already available for gambling, which constitutes an additional argument against impaired self-control in cases of gambling disorder (Grant & Potenza, 2007). Importantly, the formal diagnosis of gambling disorder may be established according to either DSM-5-TR or ICD-11 criteria (American Psychiatric Association, 2022; World Health Organization, 2025)

On the other hand, criminal behavior may be precipitated by gambling disorder, particularly in contexts of financial scarcity and desperate life circumstances among individuals without prior criminal records (Mestre-Bach et al., 2018). In this context, situations such as stealing from family members or acquaintances to obtain money to repay debts or to finance gambling activities under desperate life circumstances may be considered scenarios of partial criminal imputability (semi-imputability) under Brazilian law (Abdala-Filho et al., 2016; Mestre-Bach et al., 2018). Urgency and lack of premeditation constitute additional arguments supporting impaired self-control and partial criminal imputability in individuals with gambling disorder who engage in gambling disorder-related criminal behavior (Abdala-Filho et al., 2016; Mestre-Bach et al., 2018). Importantly, prior empirical evidence indicates that individuals with a history of gambling disorder-related criminal behavior exhibit greater disorder severity and higher levels of accumulated debt, factors that may also influence the assessment of criminal responsibility in these cases (Mestre-Bach et al., 2018). Given the multiple complexities involved in the forensic assessment of these individuals, Fig. 1 presents a conceptual model that may be useful in guiding discussions on the criminal responsibility of individuals with gambling disorder in Brazilian courts.

Nonetheless, the complex relationship between gambling disorder and criminal behavior must be evaluated on a case-by-case basis and interpreted within the framework of the Brazilian legal system. Further studies are needed to elucidate the relationship between criminal behavior and gambling disorder, including investigations into the neurobiological underpinnings, personality factors influencing this association, and the extent of self-control impairments in affected individuals (Blum & Grant, 2017; Mestre-Bach et al., 2018). In addition, research into novel psychological and pharmacological interventions aimed at offender rehabilitation is essential, including the investigation of the potential role of court-supervised treatment strategies (Blum & Grant, 2017).
